# The impact of plan complexity on calculation and measurement-based pre-treatment verifications for sliding-window intensity-modulated radiotherapy

**DOI:** 10.1016/j.phro.2024.100622

**Published:** 2024-08-05

**Authors:** Shi Li, Huanli Luo, Xia Tan, Tao Qiu, Xin Yang, Bin Feng, Liyuan Chen, Ying Wang, Fu Jin

**Affiliations:** Departments of Radiation Oncology, Chongqing University Cancer Hospital, Chongqing, Republic of China

**Keywords:** Plan complexity metric, Dosimetric accuracy, Pretreatment verifications, Algorithm, 3D diode arrays

## Abstract

**Background and purpose:**

In sliding-window intensity-modulated radiotherapy, increased plan modulation often leads to increased plan complexities and dose uncertainties. Dose calculation and/or measurement checks are usually adopted for pre-treatment verification. This study aims to evaluate the relationship among plan complexities, calculated doses and measured doses.

**Materials and methods:**

A total of 53 plan complexity metrics (PCMs) were selected, emphasizing small field characteristics and leaf speed/acceleration. Doses were retrieved from two beam-matched treatment devices. The intended dose was computed employing the Anisotropic Analytical Algorithm and validated through Monte Carlo (MC) and Collapsed Cone Convolution (CCC) algorithms. To measure the delivered dose, 3D diode arrays of various geometries, encompassing helical, cross, and oblique cross shapes, were utilized. Their interrelation was assessed via Spearman correlation analysis and principal component linear regression (PCR).

**Results:**

The correlation coefficients between calculation-based (CQA) and measurement-based verification quality assurance (MQA) were below 0.53. Most PCMs showed higher correlation *r_pcm-QA_* with CQA (max: 0.84) than MQA (max: 0.65). The proportion of *r_pcm-QA_* ≥ 0.5 was the largest in the pelvis compared to head-and-neck and chest-and-abdomen, and the highest *r_pcm-QA_* occurred at 1 %/1mm. Some modulation indices for the MLC speed and acceleration were significantly correlated with CQA and MQA. PCR’s determination coefficients (*R^2^*) indicated PCMs had higher accuracy in predicting CQA (max: 0.75) than MQA (max: 0.42).

**Conclusions:**

CQA and MQA demonstrated a weak correlation. Compared to MQA, CQA exhibited a stronger correlation with PCMs. Certain PCMs related to MLC movement effectively indicated variations in both quality assurances.

## 1 Introduction

Intensity-modulated radiotherapy (IMRT) improves target coverage and dose sparing of healthy tissue [1], and these improvements are primarily associated with an increase in beam modulation. However, the increased modulation frequently introduces greater plan complexities and dose uncertainties [2]. Consequently, it is crucial to assess the influence of plan complexity on dose [3]. On one hand, numerous metrics have been proposed to quantify plan complexity [4], [5], which can be categorized into fluence metrics, accuracy metrics, and deliverability metrics [6]. On the other hand, for pre-treatment dose verification quality assurance (QA), both dose calculation and measurement checks should be conducted following the guidelines of the American Association of Physicists in Medicine (AAPM) Task Group (TG) 219 [7].

Generally, the calculation-based QA (CQA) supports a detailed and effective investigation of a dose calculation procedure, and many algorithms are employed, including a second treatment planning system (TPS). Among them, Collapsed Cone Convolution (CCC) is commonly utilized [7], and Monte Carlo (MC) method is considered a gold standard. While the measurement-based QA (MQA) identifies uncertainties in planning and delivery systems, it is often affected by measurement and analysis tools. Noticeable differences in error sensitivity between different array geometries have been reported [8]. Cross-planar arrays are more sensitive to multi-leaf collimator (MLC) positional systematic errors and monitor unit (MU) errors than the helical array. Conversely, for collimator angle errors and MLC positional random errors, helical array demonstrates higher sensitivity than cross-planar arrays [8].

However, due to limited resources, some busy radiotherapy centers face challenges in performing MQA for every patient. Additionally, MQA has been reported to have lower sensitivity than CQA in detecting unacceptable plans [9]. However, MQA can identify delivery errors that may not be detectable with CQA alone. Moreover, these unacceptable plans are often highly complex [10], [11]. To date, numerous plan complexity metrics (PCMs) have been proposed to detect plans with large dosimetric uncertainties [4], [6]. Each PCM emphasizes distinct plan features, making it uncertain which metrics can reliably predict outcomes in CQA and/or MQA [12].

Therefore, the focus of this study was on three major aspects: the relationship between CQA and MQA, the correlation of various PCMs with QA results, and the prediction accuracy of QA results by PCMs. In the study, 53 typical PCMs were chosen and CQA was performed with both MC and CCC, while MQA was performed using 3D diode arrays with helical, cross and oblique cross geometries.

## 2 Materials and methods

### 2.1 Study design

Treatment plans were randomly assigned to two beam-matched linear accelerators (linacs) based on clinical workload. Each plan underwent MQA as part of clinical routine. A subset of these plans was then retrospectively and randomly selected for this study. CQA was performed using the same QA phantom as the MQA, avoiding different geometry effects, so any disparity is attributed to inherent differences in QA systems rather than patient anatomy. An illustrative QA flowchart and the overall study design are shown in the supplementary Fig. S1. A total of 53 PCMs were chosen [11], [15], [16], [17], [18], [19], [20], [21], [22], [23], [24], delineated in the supplementary information.

### 2.2 Patients and treatment planning

A total number of 404 sliding-window (SW) IMRT plans were collected. This study was approved by the Ethics Committee of Chongqing University Cancer Hospital (CZLS2023164-A). These plans were delivered in iX (Varian Medical Systems, Palo Alto, California, USA), including 152 cases in linac #1 (head and neck: 46, chest and abdomen: 32, pelvis: 65, limbs: 9) and 252 cases in linac #2 (head and neck: 122, chest and abdomen: 64, pelvis: 59, limbs: 7). The treatment fields, target doses, and dose constraints of organs at risk are shown in the supplementary Table S1. Plans were generated in Eclipse TPS (version 15. 6, Varian Medical Systems) and modulated with 120 leaf Millennium MLC. The dose was calculated using the Anisotropic Analytical Algorithm (AAA) with a 2.5 mm grid size, and the plan was delivered with 6-MV photon beams at 400 MU/min. Basic commissioning tests were performed according to AAPM TG 53 [13] and other international guidelines [14].

### 2.3 Measurement-based QA

Before measurement, linacs underwent mechanical and dosimetry QA to comply with AAPM TG-142 [25]. Three kinds of diode arrays were irradiated using the true composite method recommended by AAPM TG-218 [26], and each detector was calibrated for relative uniformity, absolute dose, and background correction to improve accuracy. Both helical-array ArcCHECK (Sun Nuclear Corporations, Melbourne, FL, USA) and cross-array Delta^4^ phantom+ (Delta^4+^, ScandiDos, Uppsala, Sweden) were used in linac #1, and oblique cross-array Delta^4PT^ (ScandiDos, Uppsala, Sweden) was used in linac #2.

The ArcCHECK comprises 1386 n–Si diodes arranged helically on a cylindrical PMMA surface 10.5 cm from the device center, with 1 cm center–to–center spacing between diodes, 21 cm in length and 21 cm in diameter. Data were analyzed using Sun Nuclear SNC Patient™ software (version 6.2.2). Both Delta^4+^ and Delta^4PT^ include two perpendicular planar arrays (20 × 20 cm^2^ per plane) in a cylindrical PMMA, with 1069 p–Si diodes spaced 0.5 cm apart in the central 6 × 6 cm^2^ area and 1 cm apart in the outer area. The Delta^4^ software (version 1.00.0180) was used for analysis.

Following AAPM TG-218 recommendations [26], global gamma analysis was conducted for absolute dose. Acceptance criteria of 3 %/3 mm, 3 %/2 mm, 2 %/2 mm, 1 %/1 mm, along with a dose threshold of 10 % were employed. All measured doses were not processed by any interpolation or reconstruction. All computed gamma passing rates (GPRs) were gathered for subsequent analysis.

### 2.4 Calculation-based QA

A voxel-based MC-algorithm implemented into SciMoCa (Scientific RT GmbH, Munich, Germany) was used, employing five virtual source models [27]. In our hospital, the basic beam characteristics (e.g., percent depth doses, profile curves, output factors, and dosimetric leaf gap [DLG]) of linac #1 were used to commission SciMoCa. Meanwhile, a CCC-algorithm was implemented into Mobius 3D (Varian Medical Systems, Palo Alto, California, USA), and a beam model was primarily created using a set of default beam characteristic values specified by Mobius 3D (M3D). This model was then refined by adjusting the DLG correction, with a specific value of −0.55 mm, to align with the measured dose generated by linac #2 [28].

The plans based on patients’ planning CTs were copied to CTs of two phantoms corresponding to the helical-array and the cross-array. Notably, both cross and oblique cross arrays had the same phantom. Doses was recalculated in the TPS and the plan was denoted as a QA plan. The CTs, structures and plans of the QA plans were then transferred to SciMoCa for linac #1 and M3D for linac #2. These QA plans were also utilized in the MQA process. During CQA, doses were recalculated with a 2.5-mm grid size and compared with the original dose. Similarly, all GPRs were gathered for subsequent analysis. In this document, MC-algorithm refers to SciMoCa, and CCC-algorithm refers to M3D.

### 2.5 Statistical analysis

To estimate the correlation between CQA and MQA, Spearman rank correlation coefficients (*r_calc-meas_*) were used. Additionally, the coefficients (*r_pcm-QA_*) between each metric and the GPRs of both CQA and MQA were investigated. For comparison purposes, all *r_pcm-QA_* adopted absolute values. A correlation coefficient with an absolute value of ≥ 0.7 was considered strong, 0.5–0.7 as moderate, 0.4–0.5 as weak, and < 0.4 as none. The percentage of PCMs with *r_pcm-QA_*≥0.5 (*PCMs%* ≥ 0.5) was utilized to summarize results and facilitate comparisons.

Principal component linear regression (PCR) was utilized to evaluate predictive accuracy of QA by the included PCMs. This approach includes principal component analysis and linear regression techniques to mitigate multicollinearity and bolster the model robustness. Throughout this process, major information of plan complexity was extracted from the original 53 PCMs to create new principal components for linear regression. The predictive accuracy of QA results by the combination of the PCMs can be indicated by the coefficient of determination (*R^2^*), where an *R^2^* value closer to 1 implies a larger proportion of the variance in the GPRs of QA can be explained by the PCM scores. A two-tailed *p* < 0.05 was considered statistically significant. All statistical analyses were performed using SPSS (version 26.0) software.

## 3 Results

### 3.1 The relationship between CQA and MQA

**Fig. 1:**
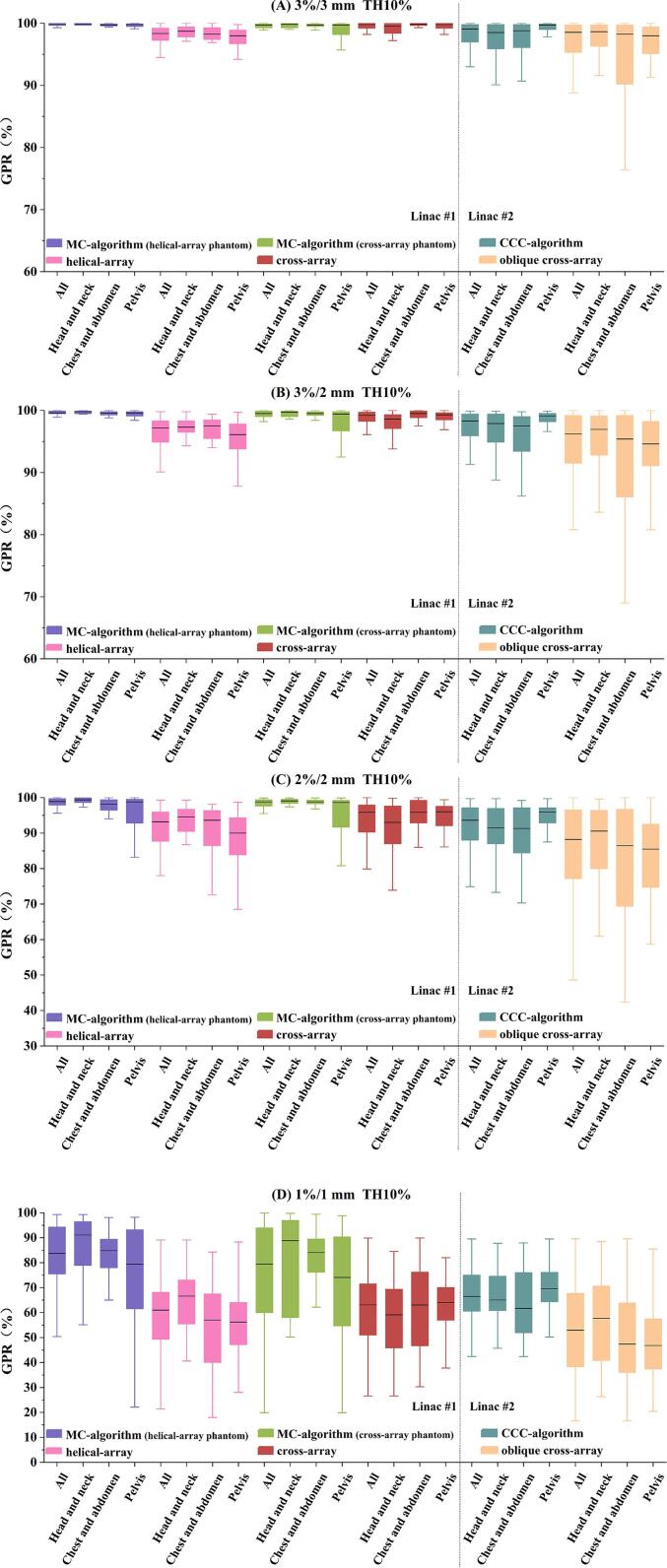
Box chart of GPRs for various QA systems corresponding to two linacs at (A) 3 %/3mm, (B) 3 %/2mm, (C) 2 %/2mm, and (D) 1 %/1mm criteria. The helical-array and cross-array were used for the same case in linac #1, the oblique cross-array was applied in linac #2. Accordingly, the MC-algorithm received the DICOM items of helical-array's and cross-array's QA plans, and the CCC-algorithm received the DICOM items of oblique cross-array's QA plans. *Abbreviations:* GPR = gamma passing rate.

A total of 4448 GPRs were collected. Fig. 1 demonstrates that across criteria from 3 %/3 mm to 1 %/1 mm, the CQA generally exhibited higher GPRs than the MQA. The disparity increased under stricter criteria. The maximum differences in median GPRs were 1.5 % at 3 %/3 mm, 2.5 % at 3 %/2 mm, 5.7 % at 2 %/2 mm, and 22.7 % at 1 %/1 mm. The *r_calc-meas_* varied among different QA systems (Table 1), ranging from 0.28 to 0.53 (*p* < 0.01) for MC-algorithm and helical-array, 0.32 to 0.42 (*p* < 0.01) for MC-algorithm and cross-array, and below 0.23 (*p* < 0.01) for CCC-algorithm and oblique cross-array. When the plans were subdivided into three treatment sites, the highest *r_calc-meas_* appeared in the head-and-neck site between MC-algorithm and helical-array, reaching 0.67 (*p* < 0.01).

**Table 1:** Spearman correlations (*r_calc-meas_*) between gamma passing rates of calculation–based QA systems and measurement–based QA systems in two linacs.

Grey highlights indicated the correlation coefficients were ≥ 0.4, **represents *p* < 0.01 and * represents 0.01 ≤ *p* < 0.05.

Fig. 1 indicates that median GPRs decreased by 0.2–2.4 % when transitioning from a 3-mm DTA to a 2-mm DTA for the same 3 % dose difference (DD). Similarly, the median GPRs decreased by 0.7 % to 8 % shifting from a 3 % DD to a 2 % DD for the same 2 mm DTA, with the lowest decrease in MC-algorithm and the highest decrease in oblique cross-array. The median GPRs of MC-algorithm exceeded CCC-algorithm, with the highest discrepancies at 1 %/1 mm. The GPRs of MQA in descending order were: cross-array, helical-array, and oblique cross-array. The performance of different QA systems varied within the same treatment site. At 1 %/1 mm, MC-algorithm, helical-array and oblique cross-array had the lowest median GPR in the pelvis compared to the other sites. Conversely, CCC-algorithm recorded the lowest median GPR in the chest-and-abdomen, while cross-array had the lowest value in the head-and-neck site.

### 3.2 Correlation of various PCMs with CQA and MQA

**Fig. 2:**
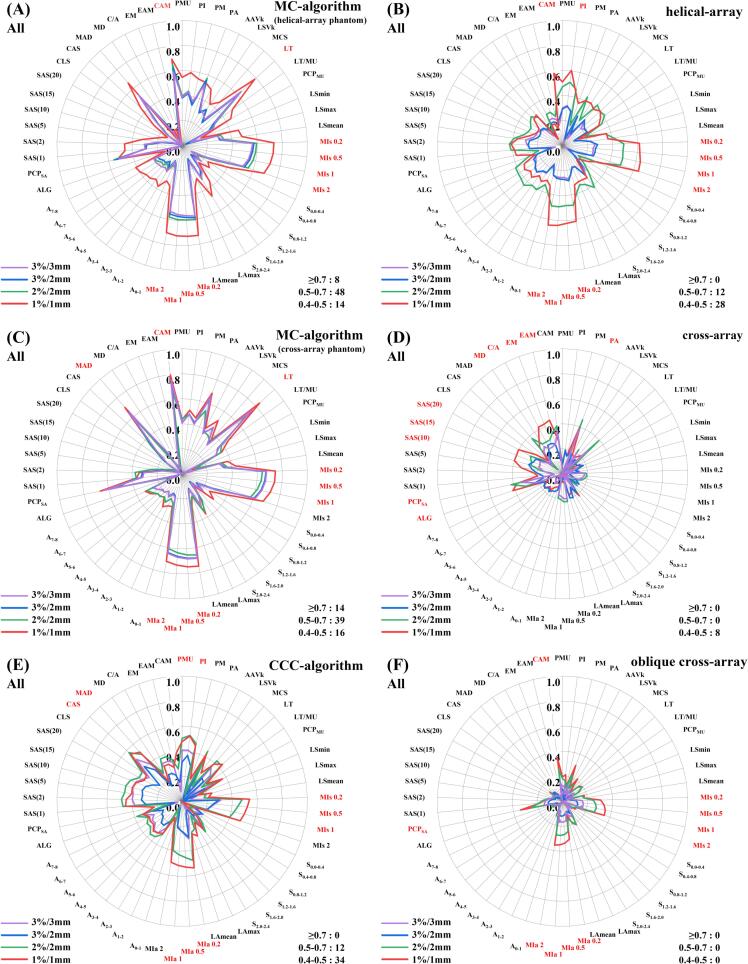
Radar–plots of Spearman correlations (*r_pcm-QA_*) between each of the studied PCM and the gamma passing rates of (A) MC-algorithm based on helical-array phantom, (B) helical-array, (C) MC-algorithm based on cross-array phantom, (D) cross-array, (E) CCC-algorithm, and (F) oblique cross-array at different criteria. The red-marked metrics represent the top 10 most relevant metrics at 1 %/1 mm criterion. (For interpretation of the references to colour in this figure legend, the reader is referred to the web version of this article.)

In Fig. 2, the *PCMs%* ≥ 0.5 ranked as follows: MC-algorithm based on helical-array, MC-algorithm based on cross-array, CCC-algorithm, helical-array, cross-array, and oblique cross-array. Moreover, the *PCMs%* ≥ 0.5 for CQA (Fig. 2A, 2C, and 2E) exceeded those for MQA (Fig. 2B, 2D, and 2F), where the maximum *r_pcm-QA_* was 0.84 (*p* < 0.01) for CQA and 0.65 (*p* < 0.01) for MQA. The radar–plot shapes formed by *r_pcm-QA_* were similar from 3 %/3 mm to 1 %/1 mm, and demonstrated larger *r_pcm-QA_* at stricter criteria, notably increasing at 2 %/2 mm and 1 %/1 mm and most prominently at 1 %/1 mm.

The higher *r_pcm-QA_* is, the better the ability is, including indicating QA variations by PCMs and recognizing the plan complexity with QA. Supplementary Table S2 grades PCMs based on their average *r_pcm-QA_*, where MI_s_ (modulation index for MLC speed), MI_a_ (modulation index for MLC acceleration), LT (leaf travel), and CAM (converted aperture metric) had the highest *r_pcm-QA_*. As depicted in Fig. 2, MI_s_ and MI_a_ consistently exhibited higher *r_pcm-QA_* compared to other PCMs (max: 0.73 for CQA and 0.65 for MQA, respectively). This trend was consistent across all QA systems except for cross-array, indicating that they could better indicate dose variations.

**Fig. 3:**
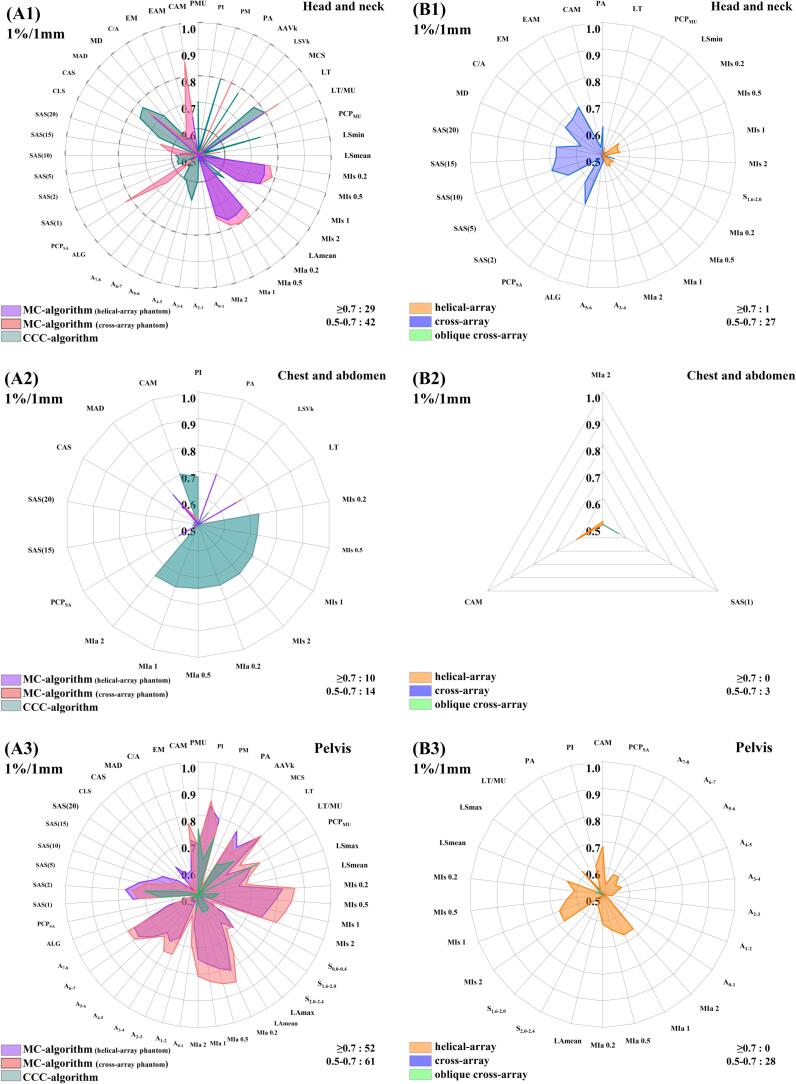
Radar–plots of Spearman correlations (*r_pcm-QA_*) between each of the studied PCMs and gamma passing rates of (A) calculation–based QA systems and (B) measurement-based QA systems at 1 %/1mm criterion in (1) Head–and−neck, (2) Chest−and−abdomen, and (3) Pelvis. At 1 %/1mm criterion, every system was associated with 53 *r_pcm-QA_*, and each treatment site comprised results from 6 systems, culminating in a total of 318 *r_pcm-QA_* per treatment site. The percentage of PCMs with *r_pcm-QA_*≥0.5 was distributed as follows: 99 (29+42+1+27)/318 for the head-and-neck, 27 (10+14+3)/318 for the chest-and-abdomen, and 141 (52+61+28)/318 for the pelvis.

Fig. 3 shows that the *r_pcm-QA_* varied considerably among three treatment sites. The *PCMs%* ≥ 0.5 was the greatest in the pelvis (141/318) compared to the other sites (99/318, 27/318). The *PCMs%* ≥ 0.5 for CQA (Fig. 3A) was greater than that for MQA (Fig. 3B). The maximum *r_pcm-QA_* for CQA was 0.86 (*p* < 0.01), while 0.7 (*p* < 0.01) for MQA. For all QA systems, LT and CAM exhibited higher *r_pcm-QA_* compared to other PCMs in the head-and-neck and the chest-and-abdomen sites, while MI_s_ and MI_a_ exhibited the highest *r_pcm-QA_* in the pelvis.

### 3.3 The predictive accuracy of QA using PCMs

The mutually independent principal components extracted from the 53 PCMs, covered over 90 % of the variance in the PCMs. In supplementary Fig. S3 (A), the *R^2^* was higher for MC-algorithm (0.56–0.71, *p* < 0.01) compared to helical-array (0.22–0.42, *p* < 0.01) and cross-array (0.25–0.42, *p* < 0.01), and CCC-algorithm had higher *R^2^* (0.29–0.40, *p* < 0.01) than oblique cross-array (0.05–0.14, *p* < 0.05) across criteria from 3 %/3 mm to 1 %/1. This trend persisted in the supplementary Fig. S3 (B-D), indicating the same PCMs exhibited higher predictability accuracy for CQA than MQA.

As shown in the supplementary Fig. S3 (B-D), MC-algorithm and helical-array showed the highest *R^2^* at 1 %/1 mm versus the other criteria. At 1 %/1 mm, the *R^2^* values of MC-algorithm were significantly higher than those of CCC-algorithm and MQA, where the maximum value was 0.92 (*p* < 0.01). Additionally, in the head–and–neck site, the *R^2^* values of cross-array were higher than those of other arrays, while in the pelvis, cross-array had the lowest *R^2^*.

## 4 Discussion

This retrospective study represents the inaugural and comprehensive investigation into the relationship between PCMs, CQA and MQA. Overall, CQA showed higher GPRs than MQA, and the *r_calc-meas_* was below 0.53, which is ascribed to several factors. Firstly, CQA and MQA entail different uncertainty sources, with CQA lacking uncertainties associated with IMRT delivery included partially in MQA. Secondly, there are inherent uncertainties associated with the QA systems, including beam modeling and algorithms for CQA, detectors for MQA, and gamma analysis tools. Hussein [29] discovered that detector array configuration and resolution significantly affected MQA results. Disparities in gamma analysis, such as interpolation algorithms, normalization techniques, contribute to inconsistencies observed across different QA systems [26].

The correlation between dose uncertainties and plan complexity was recently revealed by Mans et al. [30]. Increased complexity typically results in increased uncertainties in both planning and delivery. MQA showed weaker correlations with most PCMs, and PCMs were less predictive of MQA compared to CQA. This suggests MQA may face greater limitations on detector arrays and/or analysis methods compared to CQA, potentially masking these uncertainties. Nevertheless, CQA only used QA plans in this study, so CQA based on patient CT images will be explored in the future [27].

At 1 %/1 mm, PCMs exhibited the strongest correlation with QA, and the GPR difference between CQA and MQA increased as criteria became stricter. Employing stricter criteria enabled subtle error detection, so, the stricter metrics are more suitable for hypofractionated plans. Among the three anatomical sites, the pelvis exhibited the highest PCM percentage with *r_pcm-QA_*≥0.5 (141/318). These PCMs encompass mechanical parameters and planning properties such as MLC speed and acceleration, MU, MLC aperture. Such findings suggest that compared to other sites, the complicated pelvis plan led to the widest changes in mechanical parameters and plan properties, consequently resulting in differences in measured or calculated doses. Hence, additional vigilance is needed for the complicated pelvis plan.

LT/MU (leaf travel per MU) was highly correlated with LS_mean_ (leaf mean MLC speed) in IMRT, primarily due to the consistent fixed dose rate, making MU proportional to the delivery time. Moreover, certain metrics exhibited correlations as they dealt with similar information [12]. However, different metrics have different focuses, leading to different correlation strengths with CQA and MQA. Götstedt J et al. [10], [31] discovered that the EAM (edge area metric) has a strong correlation with dose differences. In this study, MI_s_ and MI_a_ exhibited higher correlations with CQA and most MQA. In prior research [23], a strong correlation exceeding 0.9 was found between MLC positional errors and MI_s_ and MI_a_. Because larger variations between CPs potentially increase positional uncertainties [32], MI_s_ and MI_a_ might enhance performances by putting more weight on the larger variations for MI_s_/MI_a_ calculation [23]. For example, in the case of MI_s_, a weight of 10 for a smaller variation of 0.1 cm/s, and a weight of 20 for a larger variation of 0.5 cm/s, as observed in this study.

Initially, the two linacs were beam-matched with a deviation of less than 0.5 % (e.g., percent depth doses, profile curves, and output factors) [33], and a slight difference in DLG (0.15 cm and 0.17 cm, respectively). Subsequently, treatment plans randomly assigned to two linacs were also randomly selected for the study. Moreover, the same PCMs (e.g., MI_s_, MI_a_, and MAD) demonstrated high correlations for two linacs. Finally, the PCM radar plot in the pelvis exhibited similarity between two linacs. Consequently, it was feasible to analyze results from both linacs together. Compared to MC-algorithm, CCC-algorithm (M3D) exhibited lower *r_pcm-QA_* for MI_s_, MI_a_, and MAD and smaller *R^2^*. The leading cause may be the MLC model characteristics in M3D because M3D could not ensure accurate computation for plans with diverse features using a single DLG correction value [28]. Additionally, the dose calculation algorithm could influence CQA accuracy, but its impact might be minimal in a homogeneous medium, as demonstrated in Rostami A’s study [34]. The variation in MQA system performance cannot be solely attributed to the detector shape, as evidenced by the dissimilar results obtained for cross-array and oblique cross-array, both utilizing the same cross-planar array shape. The GPR variations were larger for oblique cross-array compared to cross-array, which could be due to the latter being upgraded with a more stable detector [35], [36].

There were several limitations in the study. The PCR model exhibited poor prediction of QA results, and future improvements may come from deep learning or machine learning techniques [37], [38], [39], [40]. Additionally, this study exclusively focused on Varian Clinac iX linacs operating at 400 MU/min. Different plans and MQA methodologies were employed across the two linacs due to the retrospective nature of the analysis. The utilization of different CQA methods stemmed from the fact that each linac at our institution is equipped with a specific CQA system. These limitations will be addressed in the future. Although 53 PCMs were selected and deemed comprehensive for evaluating plan complexity, the potential emergence of new metrics should be noted as they could further enhance the evaluation process.

## Funding statement

This work was partly supported by the Chongqing Natural Science Foundation (grant no. cstc2021jcyj–msxmX0441), Chongqing medical scientific research project (Joint project of Chongqing Health Commission and Science and Technology Bureau) (grant no. 2022DBXM005), and National Natural Science Foundation of China (grant no. 11575038).

## CRediT authorship contribution statement

**Shi Li:** Methodology, Software, Resources, Data curation, Investigation, Formal analysis, Visualization, Writing – original draft. **Huanli Luo:** Conceptualization, Methodology, Software, Resources, Data curation, Writing – review & editing, Funding acquisition. **Xia Tan:** Visualization, Investigation, Formal analysis. **Tao Qiu:** Data curation, Investigation, Formal analysis. **Xin Yang:** Data curation, Writing – review & editing. **Bin Feng:** Resources, Visualization. **Liyuan Chen:** Methodology, Data curation. **Ying Wang:** Supervision, Project administration. **Fu Jin:** Conceptualization, Methodology, Supervision, Project administration, Writing – review & editing, Funding acquisition.

## Declaration of Competing Interest

The authors declare that they have no known competing financial interests or personal relationships that could have appeared to influence the work reported in this paper.
