# Supplementary material for: The impact of plan complexity on calculation and measurement-based pre-treatment verifications for sliding-window intensity-modulated radiotherapy
Source: Phys Imaging Radiat Oncol. 2024 Aug 5;31:100622. doi: 10.1016/j.phro.2024.100622 (PMC11364123; doi:10.1016/j.phro.2024.100622)
Supplement: Supplementary Data 1 [file mmc1.pdf]

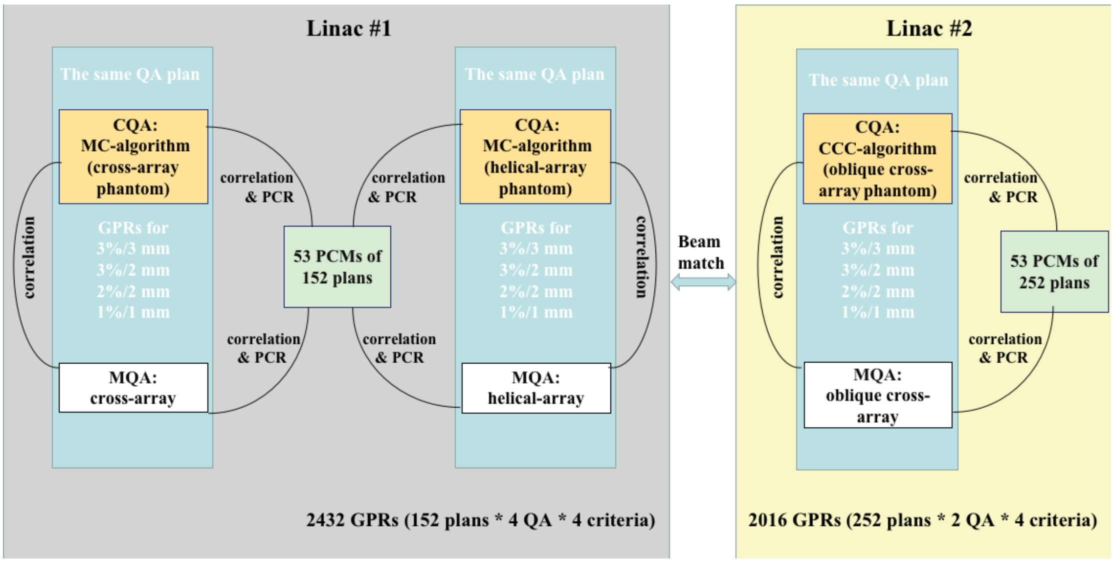

Supplementary Fig. S1. Flowchart for the application of CQA and MQA in two linacs and the study design. "The same QA plan" refers to the inclusion of the same CTs, structures, plans, and TPS dose. "Beam match" means that two linacs were beam-matched. *Abbreviations:* CQA= Calculation–based verification quality assurance; MQA= Measurement–based verification quality assurance; PCMs = Plan complexity metrics; PCR = Principal component linear regression

Supplementary Table S1. Demographics of the treatment site and dosimetric description of the sample in this study

Treatment site	Linac#1 (#Pts.)	Linac#2 (#Pts.)	Field set-up	Prescription dose	Main Dose Volume Histogram Constraints
**Head and neck**	**46**	**122**			Eye (one): Dmean<35 Gy<br>Lens (one): Dmax<6 Gy<br>Optic_Nerve (one): Dmax<54 Gy<br>Optic_Chiasm: Dmax<54 Gy<br>Brain_Stem: Dmax<54 Gy<br>Hypophysis: Dmax<54 Gy<br>Temporal_Lobes: Dmax<70 Gy<br>Thyroid: Dmean<45 Gy<br>Parotid (one): Dmean<30 Gy
Prophylactic cranial irradiation	34	7	2 opposing lateral fields	30 Gy–37.5 Gy	
Nasopharynx	12	85	9 equidistant fields	54.4 Gy–70.4 Gy	
Other	–	30			

					Oral_Cavity: Dmean<40 Gy<br>Throat: Dmean<40 Gy<br>Larynx: Dmean<40 Gy<br>Inner_ ear (one): Dmean<50 Gy<br>T-M Joint (one): V55 Gy<10%<br>Mandible: V55 Gy<10%
**Chest and abdomen**	**32**	**64**			Heart:<br>V30 Gy<40%<br>V40 Gy<30%<br>Lungs:<br>V5 Gy<60%<br>V20 Gy<30%<br>V30 Gy<20%<br>Dmean<15 Gy<br>Trachea:<br>Dmax<50 Gy<br>Stomach:<br>Dmax<50 Gy
Lung	8	6	Classic 5 fields	50 Gy−60 Gy	
Breast	3	35	4/5 tangential fields for chest wall targets and 1/2 extra small fields for supraclavicular targets	50 Gy−60 Gy	
Esophagus	7	20	butterfly−shaped 5 or 7 fields	50.4 Gy−59.4 Gy	
Other	14	3			
**Pelvis**	**65**	**59**			Small Intestine: Dmax<52 Gy<br>Bladder: V40 Gy<50%<br>Rectum: V40 Gy<50%<br>Femoral Head (one):<br>V30 Gy<30%<br>Kidney (one): Dmean<12 Gy
Cervix	50	54	7 or 9 equidistant fields	45 Gy−66 Gy	
Rectum	5	5	7 or 9 equidistant fields	45 Gy−59.4 Gy	
Other	10	−			

**Limbs**	**9**	**7**		
**All**	**152**	**252**	IMRT	Spinal_Cord: Dmax<40 Gy<br>Spinal_Cord PRV: Dmax<45 Gy

Abbreviations: Pts. = Patients

Supplementary information: Plan complexity metrics (PCMs)

A total of 53 PCMs were selected, as shown in supplementary Table S2 [2-12]. Although these PCMs addressed various plan aspects [1, 9, 11], they were approximately categorized based on their primary focus [1] for clarity and quick comprehension.

Small field features are extracted by metrics such as plan averaged beam area (PA), plan averaged beam irregularity (PI), plan averaged beam modulation (PM), percentage of control points (CPs) with segment area ＜ 5 × 5 cm^2 (PCP_{SA}), modulation degree (MD), average leaf gap (ALG), and small aperture score (SAS_x).

The deviation of the aperture from the central axis are reflected by metrics like closed leaf score (CLS), cross axis score (CAS), and mean asymmetry distance (MAD).

Circumference/area (C/A), edge metric (EM), edge area metric (EAM), and converted aperture metric (CAM) are associated with MLC edge.

Leaf minimum speed (LS_{min}), leaf maximum speed (LS_{max}), leaf mean speed (LS_{mean}), modulation index for speed (MI_s), and the average proportion of leaf speeds from a given range ($S_{l\text{–}h}$) are metrics specifically targeting the speed of MLC.

Leaf maximum acceleration (LA_{max}), leaf mean acceleration (LA_{mean}), modulation index for acceleration (MI_a), and the average proportion of leaf acceleration from a given range ($A_{l\text{–}h}$) are metrics specifically assessing the acceleration of MLC.

Leaf travel (LT) and leaf travel per monitor unit (LT/MU) are designed to evaluate leaf displacement without directly covering delivery time.

Plan normalized monitor unit (PMU) and proportion of CP with MU < 3 (PCP_{MU}) pertain to MU information.

The modulation complexity score (MCS), combining leaf sequence variability (LSVk) and aperture area variability (AAVk), is adopted to assess plan modulation level [8, 13-15].

No PCMs referring to the planning optimization was involved in this study. Notably, metrics

initially designed for arc therapy like PCP_{SA}, LT, LT/MU, LS_{min}, LS_{max}, LS_{mean}, MI_s, MI_a, A_{l-h}, S_{l-h}, LA_{mean}, LA_{max}, PCP_{MU}, MD, and EM are rendered suitable for SW IMRT. As shown in the supplementary Fig. S2, the PCMs exhibited high correlation between PCMs included, but were not limited to: LT/MU and LS_{mean}; PCP_{SA} and PA; ALG, SAS, C/A, EM, and EAM; PM, AAVk, and MCS.

Supplementary Table S2. Summary of PCMs used in this study and their QA correlation grading

Number	PCMs	Abbreviations	Reference	Correlation grading for all QA (including CQA and MQA)	Correlation grading for CQA	Correlation grading for MQA
1	Average leaf gap	ALG	[4]	√	√	√
2	Percentage of CPs with segment area <5×5cm^2	PCP_{SA}	[2]	√√	√√√	√√
3	Plan averaged beam area	PA	[5]	√√	√√√	√√
4	Small aperture score *x*=1mm	SAS(1)	[6]	√	√√	√
5	Small aperture score *x*=2mm	SAS(2)	[6]	√	√√	√
6	Small aperture score *x*=5mm	SAS(5)	[6]	√	√	√
7	Small aperture score *x*=10mm	SAS(10)	[6]	√	√	√
8	Small aperture score *x*=15mm	SAS(15)	[6]	√	√	√
9	Small aperture score *x*=20mm	SAS(20)	[6]	√	√	√
10	Closed leaf score	CLS	[6]	√	√	√
11	Cross axis score	CAS	[6]	√	√√	√
12	Mean asymmetry distance	MAD	[6]	√√	√√√	√
13	Modulation degree	MD	[3]	√	√	√
14	Segment area/Perimeter or Circumference/area	C/A	[10]	√	√	√
15	Edge metric	EM	[7]	√	√	√
16	Edge area metric	EAM	[10]	√	√	√

17	Converted aperture metric	CAM	[10]	√√√	√√√	√√
18	Plan normalized monitor unit	PMU	[5]	√√	√√√	√
19	Plan averaged beam irregularity	PI	[5]	√√	√√√	√
20	Plan averaged beam modulation	PM	[5]	√√	√√√	√
21	Aperture area variability	AAVk	[8]	√	√√	√
22	Leaf sequence variability	LSVk	[8]	√	√√	√
23	Modulation complexity score	MCS	[8]	√	√√	√
24	Leaf travel: the average distance traveled by the MLC leaves	LT	[9]	√√√	√√√√	√
25	Leaf travel per MU (mm/MU)	LT/MU	[10]	√	√√	√
26	Number of Monitor unit per Control Point and proportion of CP with MU < 3	PCP_{MU}	[2]	√	√√	√
27	Leaf minimum MLC speed	LS_{min}	[11]	√	√	√
28	Leaf maximum MLC speed	LS_{max}	[11]	√	√√	√
29	Leaf mean MLC speed	LS_{mean}	[11]	√	√√	√
30	Modulation index for MLC speed k=0.2	MI_s 0.2	[11]	√√√	√√√√	√
31	Modulation index for MLC speed k=0.5	MI_s 0.5	[11]	√√√	√√√√	√
32	Modulation index for MLC speed k=1	MI_s 1	[11]	√√√	√√√	√
33	Modulation index for MLC speed k=2	MI_s 2	[11]	√√√	√√√	√
34	The average proportion of leaf speeds from a given range (0−0.4cm/s)	$S_{0-0.4}$	[12]	√	√	√
35	The average proportion of leaf speeds from a given range (0.4−0.8cm/s)	$S_{0.4-0.8}$	[12]	√	√	√
36	The average proportion of leaf speeds from a given range (0.8−1.2cm/s)	$S_{0.8-1.2}$	[12]	√	√	√
37	The average proportion of leaf speeds from a given range (1.2−1.6cm/s)	$S_{1.2-1.6}$	[12]	√	√	√
38	The average proportion of leaf speeds from a given range (1.6−2.0cm/s)	$S_{1.6-2.0}$	[12]	√	√	√

39	The average proportion of leaf speeds from a given range (2.0−2.4cm/s)	$S_{2.0-2.4}$	[12]	√	√	√
40	Leaf maximum MLC acceleration	LA_{max}	[11]	√	√	√
41	Leaf mean MLC acceleration	LA_{mean}	[11]	√	√	√
42	Modulation index for MLC acceleration k=0.2	MI_a 0.2	[11]	√√√	√√√√	√
43	Modulation index for MLC acceleration k=0.5	MI_a 0.5	[11]	√√√	√√√√	√
44	Modulation index for MLC acceleration k=1	MI_a 1	[11]	√√√	√√√√	√
45	Modulation index for MLC acceleration k=2	MI_a 2	[11]	√√√	√√√	√√
46	The average proportion of leaf accelerations from a given range (0−1cm/s^2)	A_{0-1}	[12]	√	√	√
47	The average proportion of leaf accelerations from a given range (1−2cm/s^2)	A_{1-2}	[12]	√	√	√
48	The average proportion of leaf accelerations from a given range (2−3cm/s^2)	A_{2-3}	[12]	√	√	√
49	The average proportion of leaf accelerations from a given range (3−4cm/s^2)	A_{3-4}	[12]	√	√	√
50	The average proportion of leaf accelerations from a given range (4−5cm/s^2)	A_{4-5}	[12]	√	√	√
51	The average proportion of leaf accelerations from a given range (5−6cm/s^2)	A_{5-6}	[12]	√	√	√
52	The average proportion of leaf accelerations from a given range (6−7cm/s^2)	A_{6-7}	[12]	√	√	√
53	The average proportion of leaf accelerations from a given range (7−8cm/s^2)	A_{7-8}	[12]	√	√	√

53 PCMs were graded based on the average r_{pcm-QA} of various QA systems (3 CQA systems and 3 MQA systems) for the same metric at 1%/1 mm. $r_{pcm-QA} \geq 0.7$ was marked as √√√√, $0.5 \leq r_{pcm-QA} < 0.7$ was √√√, $0.4 \leq r_{pcm-QA} < 0.5$ was √√, and $r_{pcm-QA} < 0.4$ was √.

Supplementary Fig. S2. Spearman correlation coefficients between a subset of plan complexity metrics and their statistical significance p for the (A) linac #1 and (B) linac #2. * represents $p < 0.05$.

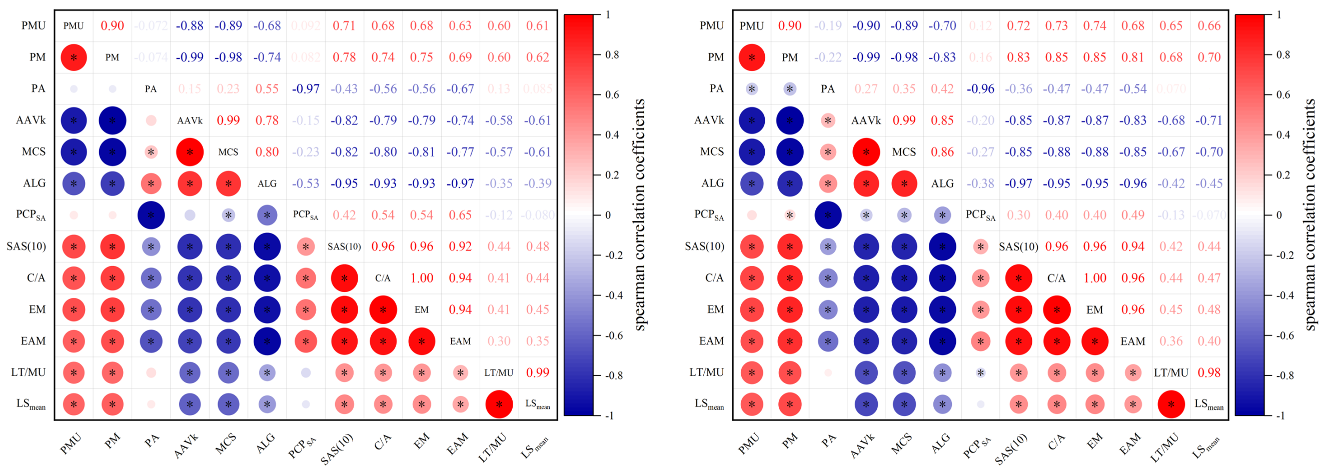

(A) Linac #1

(B) Linac #2

References

[1] Chiavassa S, Bessieres I, Edouard M, Mathot M, Moignier A. Complexity metrics for IMRT and VMAT plans: a review of current literature and applications. Br J Radiol. 2019;92:20190270.https://doi.org/10.1259/bjr.20190270.

[2] Shen L, Chen S, Zhu X, Han C, Zheng X, Deng Z, et al. Multidimensional correlation among plan complexity, quality and deliverability parameters for volumetric-modulated arc therapy using canonical correlation analysis. J Radiat Res. 2018;59:207-15.https://doi.org/10.1093/jrr/rrx100.

[3] Heijmen B, Voet P, Fransen D, Penninkhof J, Milder M, Akhiat H, et al. Fully automated, multi-criterial planning for Volumetric Modulated Arc Therapy - An international multi-center validation for prostate cancer. Radiother Oncol. 2018;128:343-8.https://doi.org/10.1016/j.radonc.2018.06.023.

[4] Nauta M, Villarreal-Barajas JE, Tambasco M. Fractal analysis for assessing the level of modulation of IMRT fields. Med Phys. 2011;38:5385-93.https://doi.org/10.1118/1.3633912.

[5] Du W, Cho SH, Zhang X, Hoffman KE, Kudchadker RJ. Quantification of beam complexity in intensity-modulated radiation therapy treatment plans. Med Phys. 2014;41:021716.https://doi.org/10.1118/1.4861821.

[6] Crowe SB, Kairn T, Kenny J, Knight RT, Hill B, Langton CM, et al. Treatment plan complexity metrics for predicting IMRT pre-treatment quality assurance results. Australas Phys Eng Sci Med. 2014;37:475-82.https://doi.org/10.1007/s13246-014-0274-9.

[7] Younge KC, Matuszak MM, Moran JM, McShan DL, Fraass BA, Roberts DA. Penalization of aperture complexity in inversely planned volumetric modulated arc therapy. Med Phys. 2012;39:7160-70.https://doi.org/10.1118/1.4762566.

[8] McNiven AL, Sharpe MB, Purdie TG. A new metric for assessing IMRT modulation complexity and plan deliverability. Med Phys. 2010;37:505-15.https://doi.org/10.1118/1.3276775.

[9] Masi L, Doro R, Favuzza V, Cipressi S, Livi L. Impact of plan parameters on the dosimetric accuracy of volumetric modulated arc therapy. Med Phys. 2013;40:071718.https://doi.org/10.1118/1.4810969.

[10] Miura H, Tanooka M, Fujiwara M, Takada Y, Doi H, Odawara S, et al. Predicting Delivery Error Using a DICOM-RT Plan for Volumetric Modulated Arc Therapy. Int J Med Phys Clin Eng Radiat Oncol. 2014;03:82-7.https://doi.org/10.4236/ijmpcero.2014.32013.

[11] Park JM, Park SY, Kim H, Kim JH, Carlson J, Ye SJ. Modulation indices for volumetric modulated arc therapy. Phys Med Biol. 2014;59:7315-40.https://doi.org/10.1088/0031-9155/59/23/7315.

[12] Park JM, Wu HG, Kim JH, Carlson JN, Kim K. The effect of MLC speed and acceleration on the plan delivery accuracy of VMAT. Br J Radiol. 2015;88:20140698.https://doi.org/10.1259/bjr.20140698.

[13] Viola P, Romano C, Craus M, Macchia G, Buwenge M, Indovina L, et al. Prediction of VMAT delivery accuracy using plan modulation complexity score and log-files analysis. Biomed Phys Eng Express. 2022;8:055020.https://doi.org/10.1088/2057-1976/ac82c6.

[14] McGarry CK, Agnew CE, Hussein M, Tsang Y, McWilliam A, Hounsell AR, et al. The role of complexity metrics in a multi-institutional dosimetry audit of VMAT. Br J Radiol. 2016;89:20150445.https://doi.org/10.1259/bjr.20150445.

[15] Li C, Tao C, Bai T, Li Z, Tong Y, Zhu J, et al. Beam complexity and monitor unit efficiency comparison in two different volumetric modulated arc therapy delivery systems using automated planning. BMC Cancer. 2021;21:261.https://doi.org/10.1186/s12885-021-07991-6.

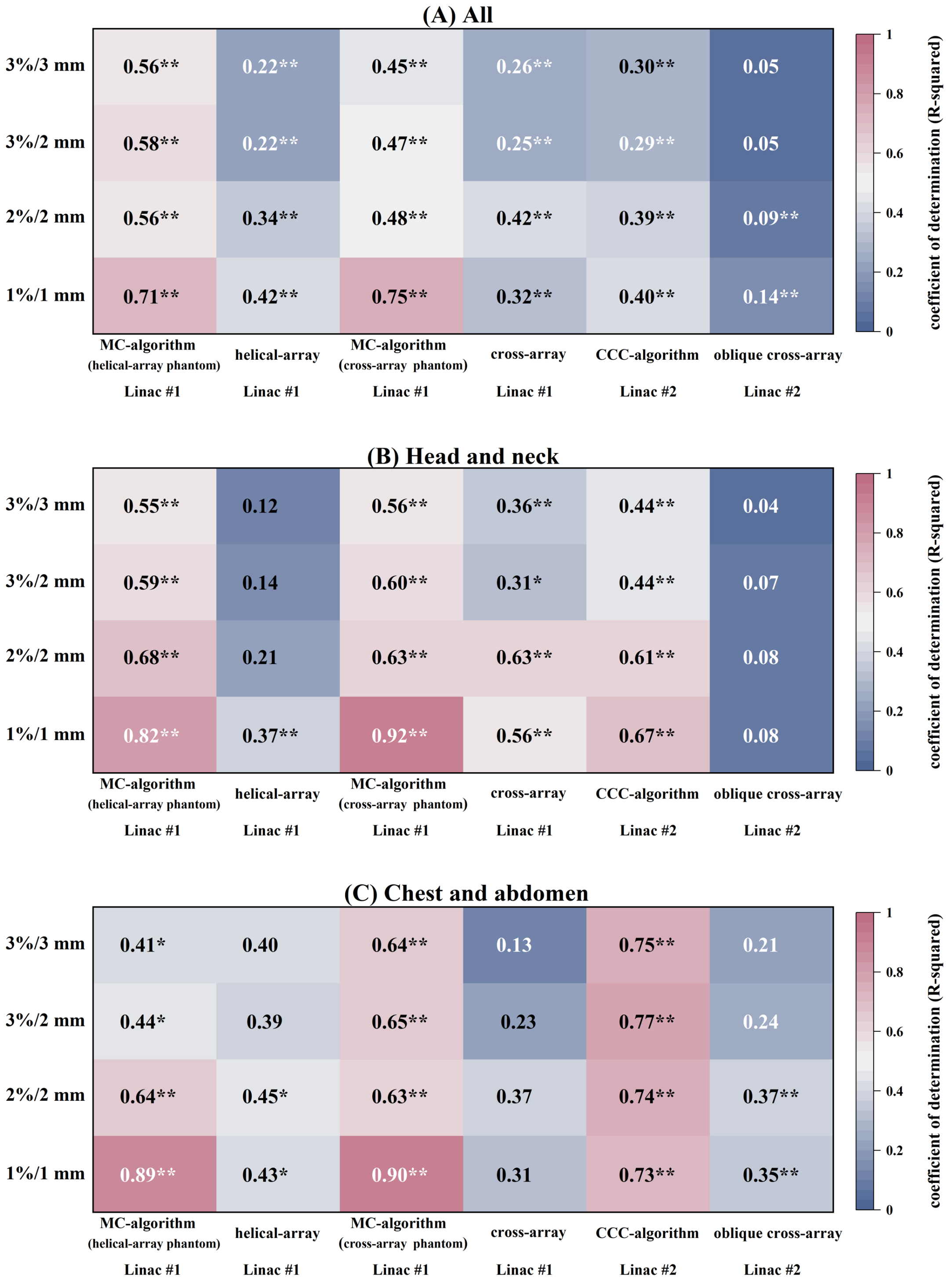
(A) All
3%/3 mm
3%/2 mm
2%/2 mm
1%/1 mm
0.56** 0.22** 0.45** 0.26** 0.30** 0.05
0.58** 0.22** 0.47** 0.25** 0.29** 0.05
0.56** 0.34** 0.48** 0.42** 0.39** 0.09**
0.71** 0.42** 0.75** 0.32** 0.40** 0.14**
MC-algorithm (helical-array phantom)
helical-array
MC-algorithm (cross-array phantom)
cross-array
CCC-algorithm
oblique cross-array
Linac #1
Linac #2
coefficient of determination (R-squared)
(B) Head and neck
0.55** 0.12 0.56** 0.36** 0.44** 0.04
0.59** 0.14 0.60** 0.31* 0.44** 0.07
0.68** 0.21 0.63** 0.63** 0.61** 0.08
0.82** 0.37** 0.92** 0.56** 0.67** 0.08
(C) Chest and abdomen
0.41* 0.40 0.64** 0.13 0.75** 0.21
0.44* 0.39 0.65** 0.23 0.77** 0.24
0.64** 0.45* 0.63** 0.37 0.74** 0.37**
0.89** 0.43* 0.90** 0.31 0.73** 0.35**
0
0.2
0.4
0.6
0.8
1

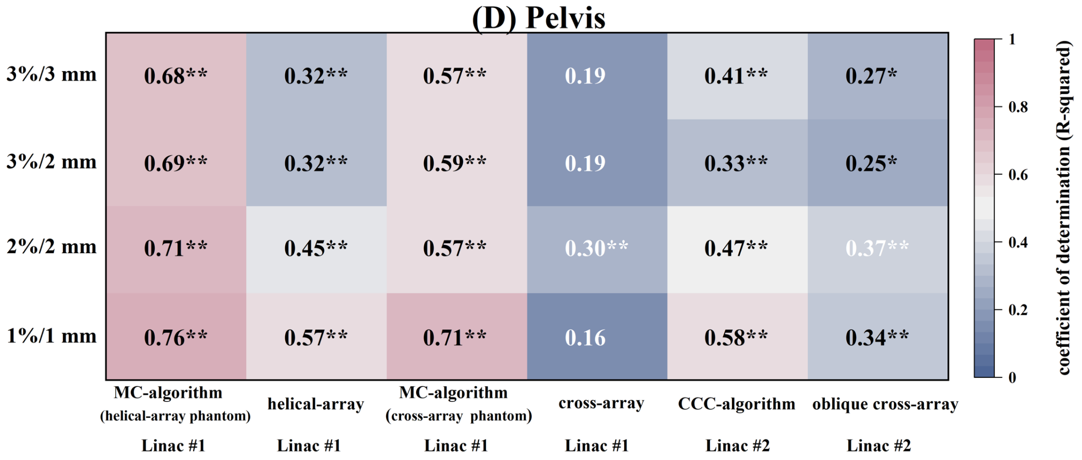

Supplementary Fig. S3. Heatmap for a coefficient of determination (R^2) of principal component linear regression using the extracted principal components factors of PCMs to explain gamma passing rates of various QA systems at 4 criteria in (A) All IMRT plans, (B) Head−and−neck, (C) Chest−and−abdomen, and (D) Pelvis corresponding two linacs. **represents $p < 0.01$ and * represents $0.01 \leq p < 0.05$.

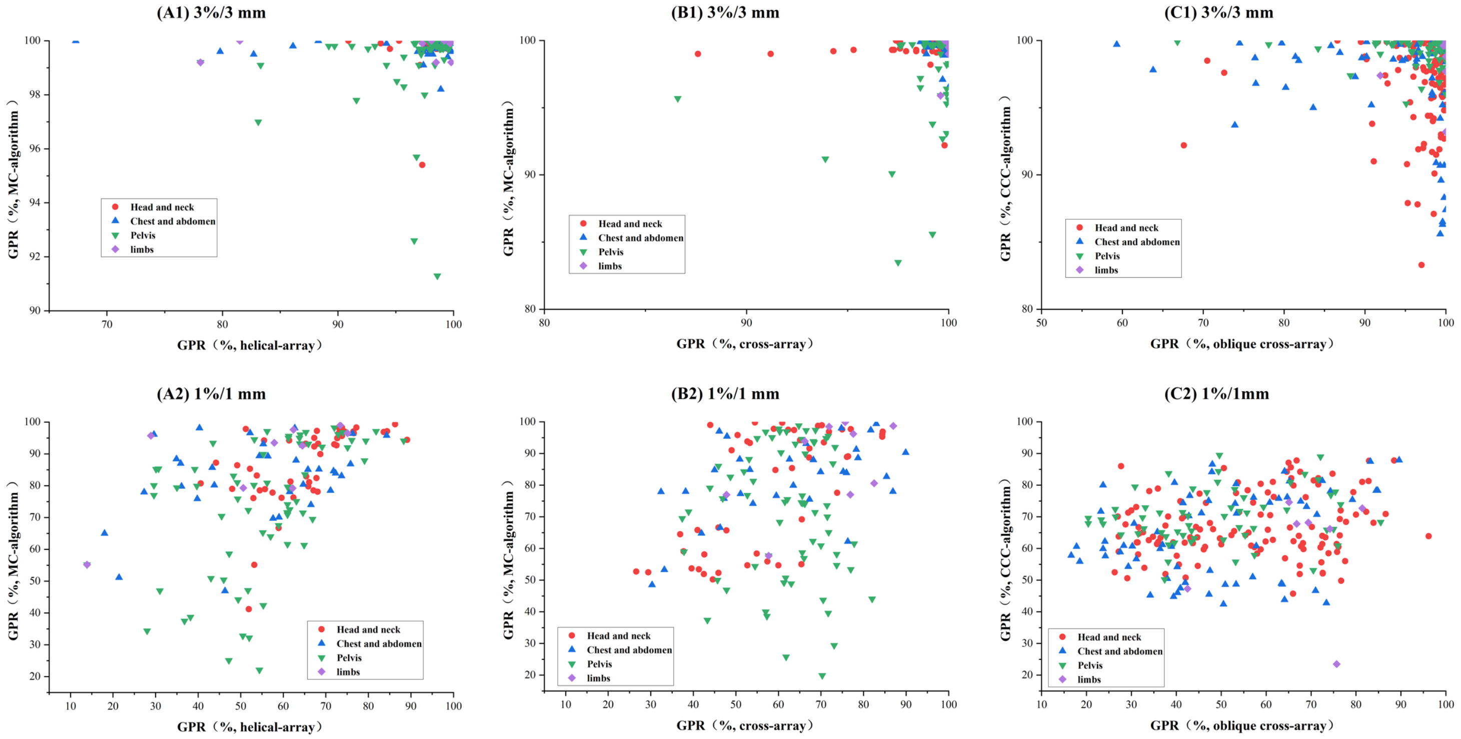

Supplementary Fig. S4. Scatter plots for gamma passing rates of calculation−based QA versus measurement−based QA at 3%/3 mm and 1%/1 mm criteria.

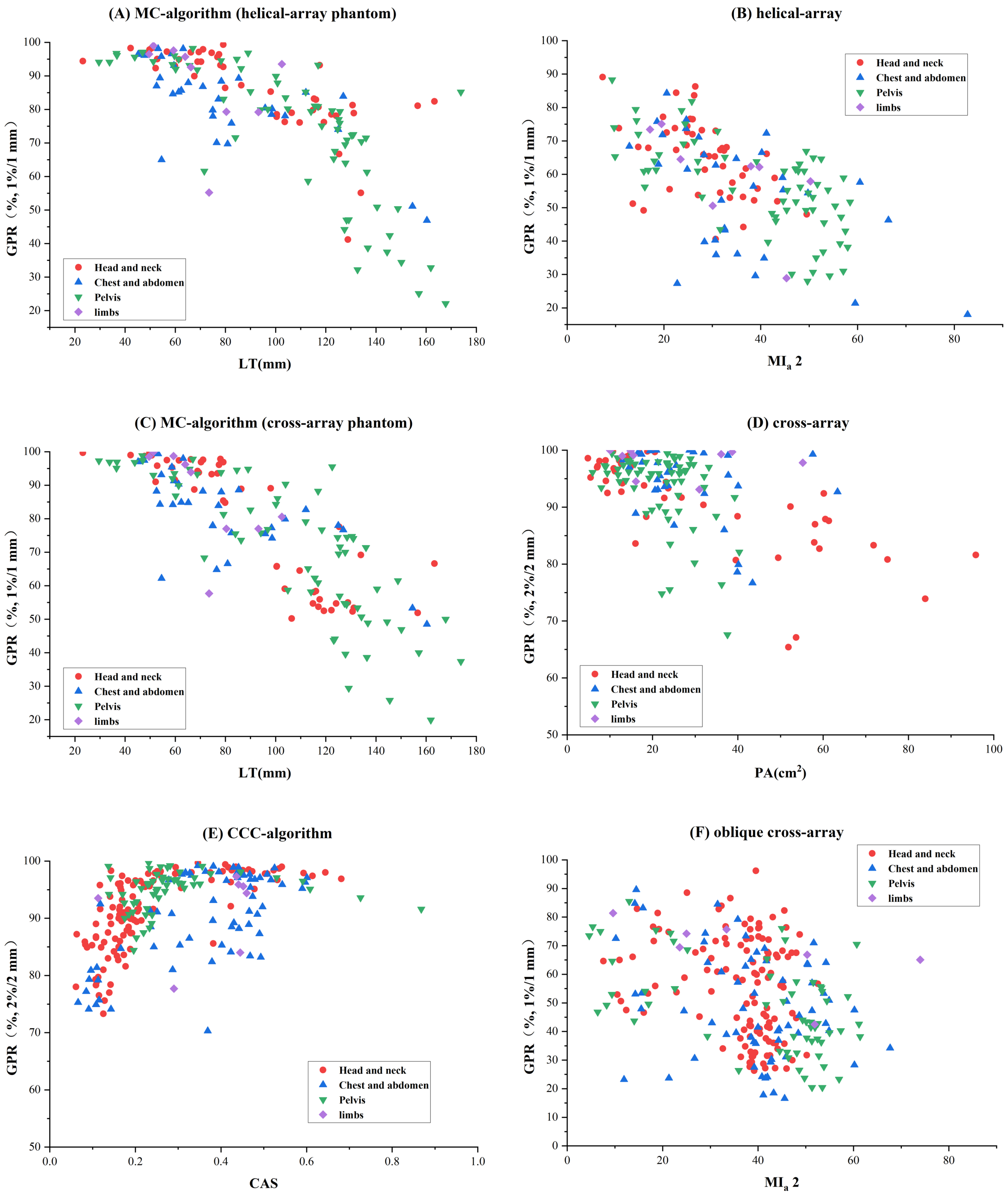

Supplementary Fig. S5. Scatter plots for gamma passing rates of QA versus the most relevant metrics at 1%/1 mm criterion.
